# CT-Based Radiomics for *a priori* Predicting Response to Chemoradiation in Locally Advanced Lung Adenocarcinoma

**DOI:** 10.3390/cancers17142386

**Published:** 2025-07-18

**Authors:** Erika Z. Chung, Laurentius O. Osapoetra, Patrick Cheung, Ian Poon, Alexander V. Louie, May Tsao, Yee Ung, Mateus T. Cunha, Ines B. Menjak, Gregory J. Czarnota

**Affiliations:** 1 Department of Radiation Oncology, Sunnybrook Health Sciences Centre, Toronto, ON M4N 3M5, Canada; erika.chung@sri.utoronto.ca (E.Z.C.); laurentiusoscar.osapoetra@sunnybrook.ca (L.O.O.); patrick.cheung@sunnybrook.ca (P.C.); ian.poon@sunnybrook.ca (I.P.); alexander.louie@sunnybrook.ca (A.V.L.); may.tsao@sunnybrook.ca (M.T.); yee.ung@sunnybrook.ca (Y.U.); 2 Physical Sciences, Sunnybrook Research Institute, Toronto, ON M4N 3M5, Canada; 3 Department of Radiation Oncology, University of Toronto, Toronto, ON M5T 1P5, Canada; 4 Evaluative Clinical Sciences, Sunnybrook Research Institute, Toronto, ON M4N 3M5, Canada; 5 Division of Medical Oncology, Sunnybrook Health Sciences Centre, Toronto, ON M4N 3M5, Canada; mateustcunha@gmail.com (M.T.C.); ines.menjak@sunnybrook.ca (I.B.M.); 6 Department of Medicine, University of Toronto, Toronto, ON M5S 3H2, Canada

**Keywords:** lung cancer, radiomics, chemoradiation

## Abstract

The standard treatment for patients with locally advanced non-small cell lung cancer (NSCLC) is concurrent chemoradiation. However, clinical responses are heterogeneous and generally not known until after the completion of therapy. Multiple studies have investigated imaging predictors (radiomics) for different cancer histologies, but little exists for NSCLC. The objective of this study was to develop a multivariate CT-based radiomics model to a priori predict responses to definitive chemoradiation in patients with lung adenocarcinoma. Methods: Patients diagnosed with locally advanced unresectable lung adenocarcinoma who had undergone chemoradiotherapy followed by at least one dose of maintenance durvalumab were included. The PyRadiomics Python library was used to determine statistical, morphological, and textural features from normalized patient pre-treatment CT images and their wavelet-filtered versions. A nested leave-one-out cross-validation was used for model building and evaluation. Results: Fifty-seven patients formed the study cohort. The clinical stage was IIIA-C in 98% of patients. All but one received 6000–6600 cGy of radiation in 30–33 fractions. All received concurrent platinum-based chemotherapy. Based on RECIST 1.1, 20 (35%) patients were classified as responders (R) to chemoradiation and 37 (65%) patients as non-responders (NR). A three-feature model based on a KNN *k* = 1 machine learning classifier was found to have the best performance, achieving a recall, specificity, accuracy, balanced accuracy, precision, negative predictive value, F1-score, and area under the curve of 84%, 70%, 80%, 77%, 84%, 70%, 84%, and 0.77, respectively. Conclusions: Our results suggest that a CT-based radiomics model may be able to predict chemoradiation response for lung adenocarcinoma patients with estimated accuracies of 77–84%.

## Simple Summary

Responses to chemoradiation can vary significantly among patients with locally advanced non-small cell lung cancer (NSCLC). The early identification of tumors that do not respond to chemoradiation is important for personalized treatment and optimized outcomes. The aim of our retrospective study was to explore CT-based radiomics as a potential way of predicting tumor response prior to the start of chemoradiation. We trained, tested, and validated a model based on the data of fifty-seven NSCLC patients. This model was able to classify tumor response with acceptable accuracy and precision. Further studies will be needed to validate the present findings.

## 1. Introduction

Despite declining incidence in recent years, lung cancer continues to be the leading cause of cancer mortality in the United States [1]. Lung cancer is categorized by histology into small-cell lung cancer and non-small-cell lung cancer (NSCLC), with the latter representing 80–85% of cases. NSCLC is further subdivided into adenocarcinoma, squamous-cell carcinoma, and large-cell carcinoma. According to the Surveillance, Epidemiology and End Results (SEER) registry, adenocarcinoma (55%) and squamous-cell carcinoma (29%) are the most common histologies [2]. Additionally, approximately 20% of NSCLC patients present with locally advanced or unresectable-stage (3A and 3B) disease [2]. Endorsed by the American Society of Clinical Oncology (ASCO) in 2015, definitive chemoradiation became the standard of care for this cohort of patients [3]. In 2018, the PACIFIC study reported that the addition of durvalumab, an immune checkpoint inhibitor, after chemoradiation improved overall survival [4]. Nonetheless, the median progression-free survival was only 5.6 and 17.2 months in the chemoradiation and chemoradiation plus durvalumab arms, respectively, underscoring the need for better treatments. These outcomes are particularly concerning given that the PACIFIC trial was already based on a heavily selected group of patients, consisting of those who did not progress after chemoradiation and, as a result, inherently had a better prognosis than those who showed disease progression and were consequently excluded from the study.

In oncology, treatment responses are extraordinarily heterogeneous. At the patient level, responses are not known until after treatment has been completed, by which time months may have been spent on futile treatments while the cancer continues to progress. If tumor response could be predicted early on or even prior to the start of treatment, patients would greatly benefit from adapted personalized approaches that optimize treatment and consequently reduce the risk of ineffective treatment. In fact, recent studies in oncology have focused on exploring ways to predict treatment response. One area being explored is radiomics, an emerging field of quantitative imaging where the phenotypic characteristics of tumors are extracted from clinical modalities (US, CT, MRI, and PET) using advanced machine learning. Our group has previously shown that non-invasive radiomics biomarkers from quantitative ultrasound can predict treatment response in patients with locally advanced breast and head and neck squamous-cell carcinoma (HNSCC) [5,6].

Published data on radiomics as a predictor for NSCLC is scant. A previous study by Ravanelli et al. reported that texture analysis on contrast-enhanced CT scans can predict responses to first-line chemotherapy for patients with advanced disease [7]. However, subgroup analyses suggested that this finding was only significant for adenocarcinoma histology. Therefore, we hypothesized that radiomics, assisted by machine learning, may also be a predictor of response in patients with locally advanced unresectable adenocarcinoma of the lung treated with contemporary chemoradiation.

## 2. Materials and Methods

### 2.1. Patient Selection

This study was approved by the Research Ethics Board at Sunnybrook Health Sciences Centre (SHSC), Toronto, Canada (ID: 034-2020). As this was a retrospective study, consent waiver was granted. Patients diagnosed with histologically confirmed locally advanced unresectable lung adenocarcinoma treated with concurrent chemoradiation followed by at least one dose of maintenance durvalumab from 2018 to 2023 were eligible for this study. Although all the patients in our cohort received consolidative durvalumab after chemoradiation, we focused only on the radiomics changes after chemoradiation to avoid potential confounding factors from the addition of durvalumab. Patients were also required to have available pre- and post-chemoradiation CT scans. Exclusion criteria included patients with large airways passing through the tumor, patients with pleural effusion where the tumor could not be discerned, and patients where the tumor encompassed the rib. Based on this set of criteria, eight patients were excluded.

### 2.2. Image Acquisition

The planning CT images were obtained using a Philips Brilliance CT Big Bore scanner with a slice thickness of 3 mm operating at 120 kVp. Slices were incremented by 1.5 mm steps. For purposes of tumor visualization, 80 mL of Omnipaque contrast medium (350 mg/mL) was injected, with the scan initiated after a calculated delay of 45 s, allowing for an optimal contrast distribution. Patient data was acquired using respiratory gating, and the maximal inspiration phase image data was used for contouring.

### 2.3. Radiomic Features

Using the CT planning scan as part of their radiotherapy planning (described above), the treating radiation oncologist contoured the gross tumor volume (GTV) to identify the tumor mass. As per departmental quality assurance protocol, all contours, including the GTV, were reviewed by the entire lung radiation oncology team (5 members). Diagnostic CT scans with intravenous contrast and radiologist reports were available for spatial corroboration to the treating radiation oncologist. The open-source Python library PyRadiomics version 3.1.0 was used to extract radiomics features from the GTV or region of interest in each patient’s radiotherapy planning scan [8]. Prior to feature extraction, we performed in-plane resampling with an in-plane spacing of 1 mm × 1 mm. The sitkBSpline was used as the interpolator. Subsequently, the image intensities were discretized into a fixed number of 32 bins for reducing the effect of noise on the extracted features and managing the computational burden. All other feature extraction settings, for instance, the wavelet settings, followed the PyRadiomics default settings. In particular, the type of wavelet used for the decomposition was “coiflets” (Coif1). The wavelet decomposition started from the original image and proceeded up to the first level, resulting in a set of wavelet decompositions {LL, LH, HL, HH}, where L refers to a low-pass filter and H refers to a high-pass filter. First-order statistical, shape/morphological, and second-order textural features were determined. The texture features include the gray-level co-occurrence matrix (GLCM) [9], gray-level run length matrix (GRLM) [10,11,12,13], gray-level size zone matrix (GLSZM) [14], neighboring gray tone difference matrix (NGTDM) [15], and gray-level dependence matrix (GLDM) [16] features. These were determined from both the original image and from the wavelet-filtered image.

Radiomic features were transformed into a standard normal distribution using the *z*-transformation. Univariate and multivariate statistical analyses were performed to evaluate the features’ ability to discriminate between a non-responder and responder. Bonferroni correction was applied to adjust the significant threshold (*p*-values < 0.0001), accounting for multiple hypothesis tests. Data cleaning and outlier removal are discussed further below.

### 2.4. Model Building and Evaluation

A binary classification problem was formulated to develop a multivariate CT-based radiomics model for predicting response to chemoradiation in patients with adenocarcinoma of the lung using a conventional machine learning approach. Comparing the diagnostic pre-treatment and post-treatment CT scans performed 1–2 months after completing chemoradiation, responses were classified into responders (R) and non-responders (NR) using the Response Evaluation Criteria in Solid Tumors (RECIST 1.1) [17]. Responders were defined as having partial and complete responses, while non-responders were defined as having stable or progressive disease.

**Figure 1:**
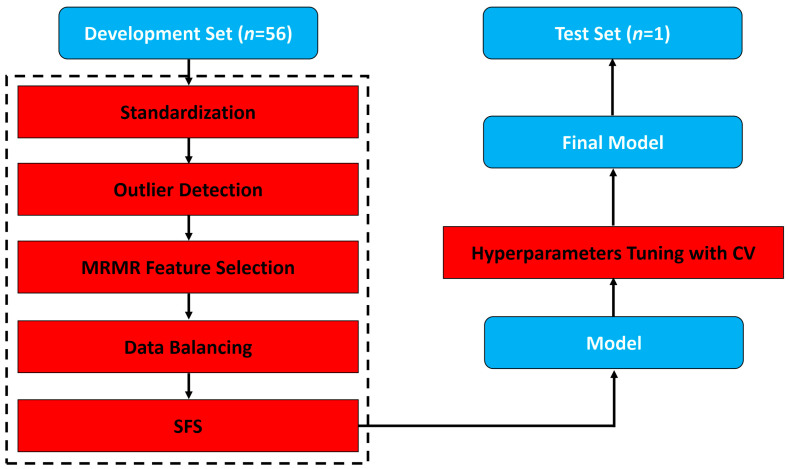
Model building and evaluation strategy. We created N external leave-one-out cross-validation (LOOCV) partitions from all samples. For each fold, we have N-1 samples for model development and a single LOO test sample, which we kept hidden for final model evaluation. From the N-1 samples, we created N-1 internal LOOCV partitions, where we fitted a classifier model on the N-2 samples and evaluated its performance on an out-of-sample LOO validation sample. We fitted N-1 models each time on different N-2 samples and eventually averaged the prediction score from all the N-1 LOO test samples. We chose the final model as the one that resulted in the highest average validation performance. The selected model then predicts the output of the previously hidden LOO test sample.

A nested leave-one-out cross-validation (LOOCV) was performed in order to build and evaluate a predictive model (Figure 1). In this procedure, one sample was set aside for independent validation in the outer fold, while the remaining data was used to train the model in the internal fold. Training consisted of several steps. First, feature standardization was applied by using the robust scalar class. After feature standardization, outliers in the training set were detected using the isolation forest technique [18]. Next, the best fifty feature subset based on the minimal redundancy–maximal relevance (MRMR) criterion [17] was selected. Following MRMR-based feature selection, the training set was balanced using the synthetic minority oversampling technique (SMOTE) [19]. The forward-sequential feature selection (SFS) method was then implemented to pick the most predictive features. Finally, grid search cross-validation was used for hyperparameter tuning.

Once the model was trained, it was validated with the previously left-out sample. The result of the validation came in the form of a prediction probability, which described the model’s confidence about the left-out sample belonging to either the responder or non-responder class. This process was repeated *N* times by leaving out a different sample for validation and using a different set of samples to develop the model. In using this approach, it was possible to evaluate the model’s ability to generalize and make predictions on new unseen data on a relatively small cohort. Once the procedure was complete, a confusion matrix was constructed, and classification metrics, including recall, specificity, accuracy, balanced accuracy, precision, negative predictive value (NPV), F1-score, and area under the curve (AUC) were reported.

## 3. Results

### 3.1. Patient Characteristics

Fifty-seven patients were ultimately included in this study after excluding patients from an initial cohort of sixty-five patients based on the exclusion criteria outlined in Section 2.1. Patient characteristics are summarized in Table 1. Almost all patients (98%) had clinical stage IIIA-C disease. The radiation dose received was 6000–6600 cGy in 30–33 fractions in all but one patient. All received concurrent platinum-based chemotherapy. The average age of patients was 66. The median pre-treatment tumor size along the longest axis was 35 mm (11–130). The median post-treatment size along the longest axis was 31 mm (8–83). According to RECIST 1.1 guidelines, 20 (35%) patients were classified as responders to chemoradiation and 37 (65%) as non-responders. These classifications were used as the ground truth labels for model evaluation.

### 3.2. Image Analysis and Data Classification

**Figure 2:**
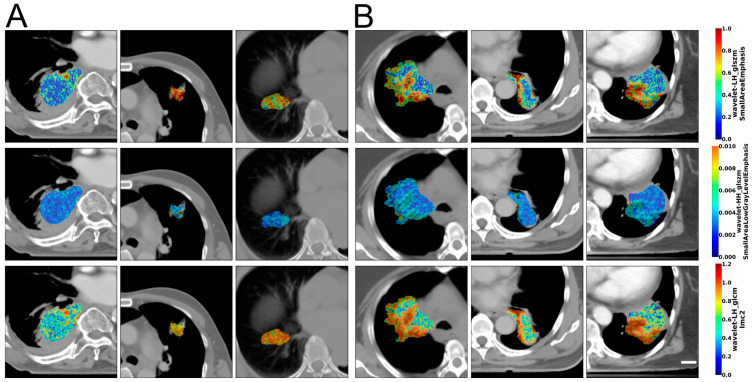
CT and texture images. Representative CT and texture images of the GTV lung nodules for (**A**) responders (left three columns showing three responder tumors) and (**B**) non-responders (right three columns showing three non-responder tumors). These include GLSZM small-area emphasis (from a wavelet LH-filtered image), GLSZM small-area low-gray-level emphasis (from a wavelet HH-filtered image), and GLCM-IMC2 (from a wavelet-LH filtered image) features. These constituted the three-feature KNN *k* = 1 model that resulted in the best generalization performance when evaluated on the LOO test samples. The scale bar represents 2 cm.

Figure 2 illustrates representative CT and texture images of the GTV lung nodules from three responders and three non-responders. The color overlays represent texture features which prominently display differences between responder and non-responder patients. The texture maps include GLSZM small-area emphasis (from a wavelet LH-filtered image), GLSZM small-area low-gray-level emphasis (from a wavelet HH-filtered image), and GLCM-IMC2 (from a wavelet LH-filtered image) features. These constituted the three-feature KNN *k* = 1 model that resulted in the best generalization performance when evaluated on the LOO test samples.

**Figure 3:**
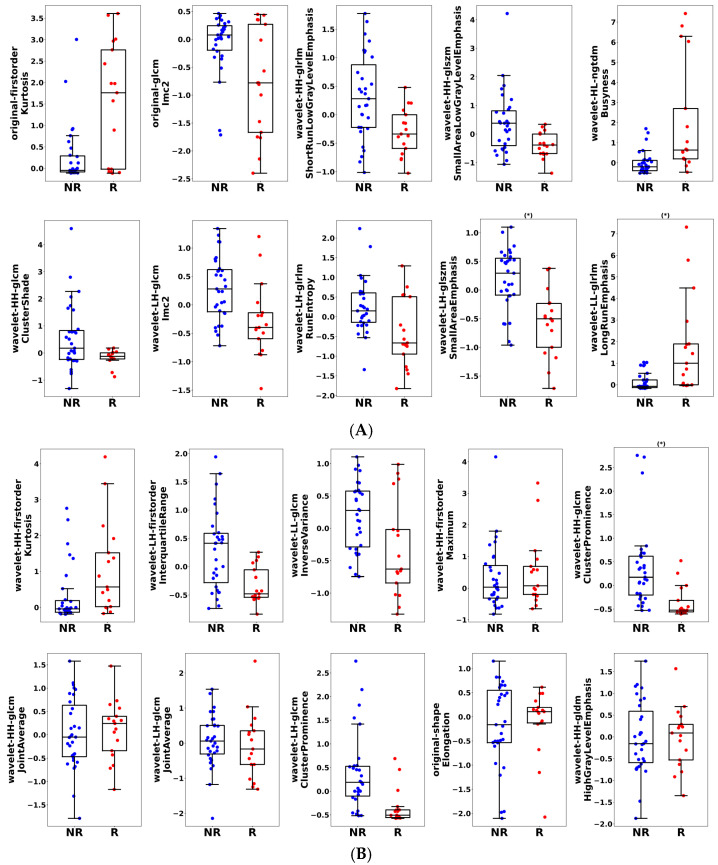
(**A**) Representative box and scatter plots of features mostly selected for the optimum KNN model. Across 57 nested LOO folds, we identified features that were selected for building a model. The presented features were transformed into standard normal distribution using the z-transformation. A univariate statistical analysis of these features indicated statistically significant differences (*p*-values < 0.0001) between non-responders (NR) and responders (R). Bonferroni correction was applied to adjust the significant threshold, accounting for multiple hypothesis tests. Although the univariate analysis indicated that not all features were statistically significant, we still can appreciate the separation between the distributions of feature values for the two groups. A multivariate combination of these features can still lead to a predictive model with decent generalization performance. (*) indicates statistically significant differences between responder and non-responder patients (*p* < 0.05). (**B**) Representative box and scatter plots of features that were not selected for the optimum KNN model. Across 57 nested LOO folds, we identified features that were not selected for building a model. The presented features were transformed into standard normal distribution using the z-transformation. Although the univariate analysis showed that a few of these features demonstrated statistically significant differences (*p*-value < 0.0001) between non-responders (“NR”) and responders (“R”), most of the features showed a clear overlap between the distribution of values among the two groups. (*) indicates statistically significant differences between responder and non-responder patients (*p* < 0.05).

Figure 3 presents representative box and scatter plots of features mostly selected for the optimum KNN (Figure 3A) and not selected for the optimum KNN (Figure 3B).

Among the selected features, parameters related to “small-area emphasis” (the extent to which an image shows a high concentration of pixels with similar intensity values clustered in small regions) and parameters related to “long-run emphasis” (indicating the presence of long stretches of pixels with the same intensity value), signifying a more coarse texture within the image, were different between responder and non-responder patients. Features not selected included wavelet-based parameters, which included their mean and variance.

**Figure 4:**
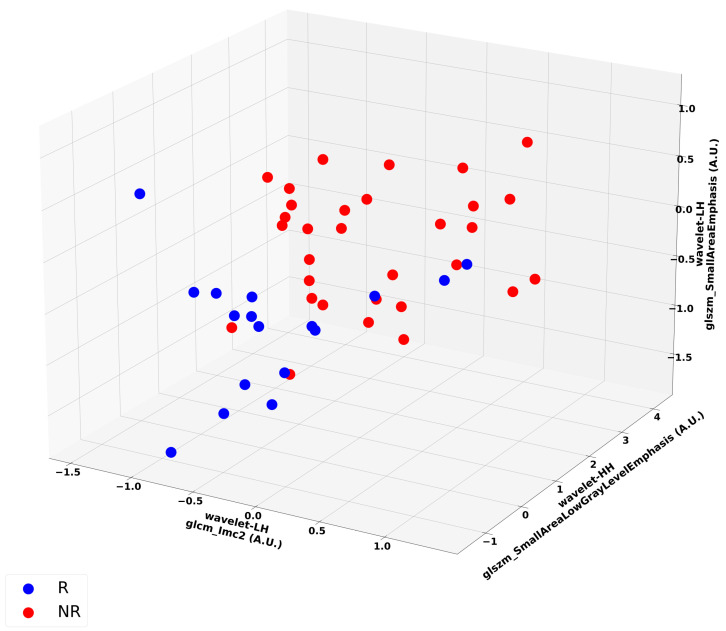
Representative scatter plots for the optimal three-feature KNN model. The features included were wavelet_LH_glszm_SmallAreaEmphasis, wavelet_HH_glszm_SmallAreaLowGrayLevelEmphasis, and wavelet-LH_glcm_Imc2. Blue dots indicate responders (“R”), while red dots indicate non-responders (“NR”). Separability between the two clusters corresponding to the responders and the non-responders, respectively, in the three-dimensional feature space is evident (dashed line).

Figure 4 is a representative scatter plot for the optimum three-feature KNN model. The best performing classification model from the LOOCV analysis was a three-feature model using the KNN classifier. Table 2 and Table 3 summarizes the classification results of the test and validation performance metrics. Overall, this model achieved 84% recall, 70% specificity, 80% accuracy, 77% balanced accuracy, 84% precision, 70% NPV, an 84% F1-score, and 0.77 AUC. There was evident separability between responder and non-responder patients.

Accuracies in the test-set ranged from 63 to 80% amongst the various models (with balanced accuracies slightly lower), and in the validation set, accuracies ranged from 82 to 88%. The F1-score of 84% for the best classifier represents an accuracy taking into account the effects of data imbalance.

## 4. Discussion

Radiomics has been explored for predicting treatment response in many different cancer histologies, utilizing a range of imaging modalities such as ultrasound, CT, MRI, and PET. Imaging-based models that can accurately predict future responses are valuable for personalized radiotherapy modification, as they can guide dose de-escalation for responders, thereby reducing toxicity for organs at risk (OARs), or prompt palliative schedules or alternative treatment regimens for non-responders. The work here represents the first CT-based radiomics study to predict treatment response in patients with locally advanced unresectable adenocarcinoma of the lung. More importantly, the study identified that a CT-based radiomics model may have prognostic value for lung adenocarcinoma patients.

We limited the study focus here to adenocarcinoma histology rather than all different histologies in NSCLC. The reasoning was three-fold. First, Ravanelli et al. found that texture analysis was a significant predictor of treatment response to first-line chemotherapy for patients with adenocarcinoma but not in those with squamous-cell carcinoma [7]. Second, different NSCLC histologies may differ in behavior and response, making it logical to focus the analysis on a single histology. Third, according to the SEER registry, adenocarcinoma is now the most common histology for NSCLC, making it the most clinically relevant NSCLC subtype to study [2].

Most lung radiomics studies have looked at ultrasound. This study evaluated CT, as it is readily available and used commonly in staging, radiotherapy planning, and evaluating treatment responses of NSCLC. Specifically, the radiomics model derived here exhibited promising classification results and may serve as a platform for further studies, such as testing whether there are differences between different patient characteristics, histologies, or PD-L1 expression levels on image characteristics.

In the phase 3 RTOG 9410 trial, patients with unresectable stage II-III NSCLC were randomized to sequential or concurrent chemotherapy and radiotherapy [20]. The concurrent chemoradiation arm with radiation delivered in conventional fractionation had a WHO-defined response rate of 70% (complete and partial response, ≥50% decrease) and a clinical complete response of 42%. In the PACIFIC study of the addition of durvalumab, the complete and partial response in the chemoradiation-only arm was 3% and 47%, respectively, as measured by RECIST 1.1 [4]. The differences between RTOG 9410 and PACIFIC may be attributed to different chemotherapy regimens and different response classification criteria. The work here used RECIST 1.1 to define responders versus non-responders. It was found that 35% of patients were responders, which appears to be lower than the PACIFIC study. This difference may be due to the RTOG 9410 and PACIFIC trials including all NSCLC histologies, whereas the study here evaluated only adenocarcinoma.

Previous studies have utilized Positron emission tomography (PET) metabolic parameters, such as the maximum standardized uptake value (SUV_max_), to predict radiological response in NSCLC [21]. Additionally, radiomics analysis on PET images has resulted in PET-based texture and heterogeneity parameters that are associated with RECIST response [21,22]. In contrast, the current study focuses specifically on radiomics features derived from baseline CT scans to predict radiological response without incorporating PET data.

Prior research on the use of radiomics of CT images to predict treatment response in NSCLC typically falls into two categories: studies that use pathological endpoints and those that utilize radiological endpoints to assess treatment outcomes [23]. Pathological response evaluation utilizes surgical pathology reports to identify three categories of pathological response, namely pathological complete response (pCR), microscopic residual disease (MRD), or gross residual disease (GRD) [24]. Radiological response, on the other hand, assesses tumor responses with the Response Evaluation Criteria in Solid Tumours (RECIST). Based on these pathological response categories, two classifications of treatment response are created, including responders to neoadjuvant chemoradiation, defined as pCR versus non-pCR (including MRD and GRD), and poor responders, defined as GRD versus non-GRD (including pCR and MRD) [24].

Coroller et al. evaluated the utility of radiomics features in predicting pathological responses in NSCLC [24]. Among 127 participants, they identified seven features predictive of GRD (*p* < 0.05) and one predictive of pCR (*p*-value < 0.05), while conventional imaging features (tumor volumes and diameters) were not predictive [24]. In a separate study of 85 participants with resectable locally advanced NSCLC, they identified three radiomics features from the primary tumors and lymph nodes as significant predictors of pCR (AUC = 0.67) [25]. Additionally, two lymph node features were predictors of GRD (AUC = 0.72–0.75) [25]. A multivariate analysis showed that combining radiomic and clinical features improved GRD classification, while radiomic features alone were optimal for pCR classification [25]. Chong et al. quantified first-order statistical CT features in two cohorts of NSCLC participants receiving a combination of concurrent chemoradiotherapy (CCRT) and tyrosine kinase inhibitor (TKI) therapy prior to surgical resection [26]. They identified kurtosis predictive of pathological response in the CCRT group, while intensity variability was predictive in the TKI group [26].

Ravanelli et al. performed texture analysis to predict radiological response in NSCLC to systemic therapy [7]. Among 53 participants, they identified gray-level uniformity predictive of RECIST response in the adenocarcinoma subgroup [7]. Yang et al. evaluated the effectiveness of radiomics features to predict radiological response in NSCLC [27]. Among 322 participants, they built a six-feature model that achieved a 0.746 AUC on separate validation data [27]. Furthermore, several studies have evaluated radiomics features to predict tumor shrinkage in NSCLC [28,29]. Hunter et al. developed a regression model to predict a reduction in tumor sizes using geometry, intensity histogram, absolute gradient, GLRLM, and GLCM features [28]. Subsequently, Ramella et al. evaluated the utility of radiomics features in predicting radiological responses in NSCLC [29]. In a cohort of 91 participants, seven radiomic features and five conventional clinical features were predictive of tumor volume after the completion of CCRT [29]. Although tumor shrinkage indicates response to treatment, further stratification based on the tumor size reduction indicated in RECIST can further improve the utility of this prediction model in predicting radiological responses. The best model reported by Hunter et al., for example, only identified samples with a reduction in tumor size approximately greater than or equal to 50% or above (GTV_6_weeks/GTV_planning ~> 50%) [28]. Therefore, their prediction model did not consider any non-responder samples, which are important ones to be identified for potentially personalized treatment adjustments.

Among the evaluated machine learning classifiers in this study, the KNN *k* = 1 model achieved optimal generalization performance of 84% recall, 70% specificity, 80% accuracy, 77% balanced accuracy, and 0.79 AUC on the LOO hold-out test samples. The KNN *k* = 3 model obtained similar generalization performance with 81% recall, 70% specificity, 77% accuracy, 76% balanced accuracy, and 0.78 AUC. SVM-Linear followed with 78% recall, 65% specificity, 74% accuracy, 72% balanced accuracy, and 0.71 AUC. Model complexity analysis was performed by varying the number of selected features to build the model. Analysis indicated that the optimum model constituted three features. Specifically, the KNN *k* = 1 model comprised wavelet LH GLSZM small-area emphasis, wavelet HH GLSZM small-area low-gray-level emphasis, and wavelet LH GLCM IMC2 features. These features were textural features derived from the wavelet decomposition of the original images. Wavelet decomposition transforms complex image data into different frequency bands, revealing distinct features and patterns not readily visible in the original image. Sequential decomposition, through the application of a series of low-pass (L) and high-pass (H) filters, extracts low-frequency components (LL) and high-frequency components (LH, HL, and HH), respectively. The characterization of heterogeneity in the high-frequency wavelet-filtered images of the lung nodule appears to be most important in producing the optimum model.

Since determined quantitative imaging features can be affected by many factors, it is necessary to perform comparative analysis among different radiomics methodologies. The image biomarkers standardization initiative (IBSI) aimed to address the standardization of the extraction of these features [30]. Standardization will ease comparative analysis and allow for reproducibility analysis among different radiomics studies analyzing the same effect. Lei et al. performed a benchmark study of various radiomics software programs including PyRadiomics, Medical Imaging Interaction Toolkit (MITK), LIFEx, Standardized Environment for Radiomics Analysis (SERA), Cancer Imaging Phenomics Toolkit (CaPTk), and a MATLAB library from McGill University and an in-house MATLAB library [31]. They evaluated the feature agreement of these different radiomics software programs and identified that while most first-order and textural features showed satisfactory agreement, morphological features exhibited significant variation [31]. In this study, the PyRadiomics platform was only used for feature extraction.

Our study focused on the radiomics changes after chemoradiation for locally advanced adenocarcinoma of the lung. Although all patients received consolidative durvalumab afterwards, we did not look at its potential radiomics changes because it would be challenging to accurately attribute any changes to either the chemoradiation and/or durvalumab components. Recently, several studies reported on CT-based radiomics as a predictor of efficacy outcomes after immunotherapy [32,33]. Yolchuyeva et al. recently found that combined pre-treatment CT-based radiomics and clinical variables could predict overall survival and progression-free survival to nivolumab or pembrolizumab, both immune checkpoint inhibitors, in metastatic NSCLC [32]. Preliminary data from De Miguel-Perez’s group found that the combination of pre-treatment CT-based radiomics and plasma extracellular vesicle PD-L1 appeared to predict responses to pembrolizumab in metastatic NSCLC [33]. The LECOMTE-PACIFIC study found that combining clinical variables and CT-based radiomics, reticulation volume/total lung capacity and lung volume, obtained prior to chemoradiation and immunotherapy in the experimental arm of the PACIFIC, predicted developing symptomatic pneumonitis [34].

A limitation of this study is the relatively small sample size, though a well curated data set in which tumor contours were placed and agreed upon at a quality analysis step by all three radiation oncologists involved in the treatment of lung cancers at the performing institution was used. Due to the small patient cohort, there were not enough tumor cases for size-based stratification in the present study, but this option will certainly be considered in the future. In addition, an external test cohort was not available. Future studies will assess the generalizability of the proposed radiomics-based model on a larger cohort and on multi-center data. Incorporating test–retest analysis on a larger cohort to evaluate the stability of the radiomics features, which has been performed in numerous past studies, also stands as a priority. Furthermore, we approximated the characteristics of the tumors using in-plane features volumetrically. Future studies include developing a radiological response prediction model using full 3D radiomics features. However, non-isotropic image resolution across all three dimensions may pose a potential confounding factor. Additionally, based on our experience with radiomics-based models for diagnostic and prognostic applications, combining clinical and imaging features has generally been found to improve generalization performance. Therefore, we expect a similar trend in this case and will devote the investigation of clinical features to a future study.

## 5. Conclusions

This study demonstrates the robustness of CT-based radiomics framework for evaluating radiological response in NSCLC tumors. By employing a conventional machine learning approach, a reliable and well-performing classification model was developed. The analysis adhered to stringent guidelines for model development and evaluation, ensuring proper estimation of the model’s generalizability. These findings underscore the potential of CT radiomics as a valuable imaging adjunct for the *a priori* prediction of treatment response, ultimately supporting more personalized and effective treatment strategies.

## Figures and Tables

**Table 1:** Patient and treatment characteristics.

Characteristic	N = 57
Median age, years (range)	66 (41–81)
Sex, no. (%)	
Male	24 (42%)
Female	33 (58%)
Smoking status, no. (%)	
Never smoked	6 (11%)
Current/former smoker	47 (83%)
Exposed to second-hand smoke	4 (7%)
Disease stage, no. (%)	
3A	24 (42%)
3B	27 (47%)
3C	5 (9%)
4A	1 (2%)
Median initial tumor size, mm (IQR)	35 (26–60)
Median residual tumor size, mm (IQR)	31 (20–42)
PD-L1 expression level, no. (%)	
<1%	15 (26%)
1–49%	13 (23%)
≥50%	27 (47%)
Not reported	2 (4%)
EGFR (%)	
Negative	27 (47%)
Positive	13 (23%)
Indeterminate	15 (26%)
Not reported	2 (4%)
ALK-1 (%)	
Negative	36 (63%)
Positive	4 (7%)
Not reported	17 (30%)
Radiotherapy dose fractionation, cGy/# of fractions (%)	
6000/30	37 (65%)
6600/33	19 (33%)
4500/25 + SBRT boost	1 (2%)
Concurrent chemotherapy regimen (%)	
Cisplatin, etoposide	16 (28%)
Carboplatin, paclitaxel	7 (12%)
Carboplatin, pemetrexed	12 (21%)
Cisplatin, pemetrexed	22 (39%)
Response group based on RECIST 1.1, no. (%)	
Non-responder (stable/progressive disease)	37 (65%)
Responder (partial/complete)	20 (35%)

**Table 2:** Test performance.

Classifier	Recall (%)	Specificity (%)	Accuracy (%)	Balanced Accuracy (%)	Precision (%)	NPV (%)	F1-Score (%)	AUC
LDA	78	50	68	64	74	56	53	0.66
KNN *k* = 1	84	70	80	77	84	70	84	0.77
KNN *k* = 3	81	70	77	76	83	67	68	0.78
KNN *k* = 5	76	60	70	68	78	57	59	0.69
SVM-Linear	78	65	74	72	81	62	63	0.71
Random Forest	68	55	63	61	74	48	51	0.65
XGBoost	68	55	63	61	74	48	51	0.65

**Table 3:** Validation performance.

Classifier	Recall (%)	Specificity (%)	Accuracy (%)	Balanced Accuracy (%)	Precision (%)	NPV (%)	F1-Score (%)	AUC
LDA	87 ± 5	78 ± 7	82 ± 3	83 ± 3	81 ± 5	87 ± 4	81 ± 4	0.86 ± 0.04
KNN *k* = 1	84 ± 6	93 ± 4	88 ± 3	88 ± 3	93 ± 4	87 ± 4	89 ± 3	0.88 ± 0.03
KNN *k* = 3	81 ± 5	94 ± 4	88 ± 4	88 ± 4	94 ± 4	84 ± 4	89 ± 3	0.92 ± 0.04
KNN *k* = 5	78 ± 7	94 ± 5	86 ± 3	86 ± 3	94 ± 5	83 ± 5	87 ± 3	0.91 ± 0.04
SVM-Linear	86 ± 6	78 ± 8	82 ± 3	82 ± 3	82 ± 6	86 ± 4	81 ± 4	0.78 ± 0.17
Random Forest	82 ± 6	87 ± 7	85 ± 5	85 ± 5	88 ± 6	84 ± 4	85 ± 5	0.91 ± 0.03
XGBoost	80 ± 7	86 ± 7	83 ± 5	83 ± 5	87 ± 6	82 ± 5	83 ± 5	0.89 ± 0.04

## Data Availability

The datasets used and/or analyzed during the current study are available from the corresponding author upon reasonable request.

## Abbreviations

The following abbreviations are used in this manuscript:

NSCLC	Non-small-cell lung cancer
US	Ultrasound
CT	Computed tomography
MRI	Magnetic resonance imaging
PET	Positron emission tomography
PD-L1	Programmed cell death ligand-1
EGFR	Epidermal growth factor receptor
ALK-1	Activin receptor-like kinase-1
SEER	Surveillance, Epidemiology and End Results
RECIST	Response Evaluation Criteria in Solid Tumors
kVp	Kilovoltage peak
GLCM	Gray-level co-occurrence matrix
GRLM	Gray-level run length matrix
GLSZM	Gray-level size zone matrix
IQR	Interquartile range
NGTDM	Neighboring gray tone difference matrix
GLDM	Gray-level dependence matrix
LOOCV	Leave-one-out cross-validation
LOO	Leave one out
MRMR	Minimal redundancy–maximal relevance
SMOTE	Synthetic minority oversampling technique
SFS	Forward-sequential feature selection
NPV	Negative predictive value
AUC	Area under the curve
LDA	Linear discriminant analysis
KNN	K-nearest neighbors
SVM-Linear	Linear support vector machines
SVM-RBF	Support vector machines—radial basis function
RF	Random forest
XGBoost	Extreme gradient boosting
IMC2	Informational Measure of Correlation 2
AU	Arbitrary unit
pCR	Pathological complete response
MRD	Microscopic residual disease
GRD	Gross residual disease
IBSI	Image biomarkers standardization initiative
RTOG	Radiation therapy oncology group
CCRT	Chemoradiotherapy
TKI	Tyrosine kinase inhibitor
MITK	Medical Imaging Interaction Toolkit
SERA	Standardized Environment for Radiomics Analysis
CaPTK	Cancer Imaging Phenomics Toolkit
